# Trust, Cultural Competence, and Promotores-Based Engagement: Effective Outreach with Dementia Family Caregivers

**DOI:** 10.1093/geroni/igaf122.2861

**Published:** 2025-12-31

**Authors:** Alma Manzo, Raheleh Bahrami, Maria Gonzalez Pyles, Lourdes Cordova, Berta Carbajal, Allison Glinka, David Coon

**Affiliations:** Arizona State University, Phoenix, Arizona, United States; Arizona State University, Phoenix, Arizona, United States; Arizona State University, Phoenix, Arizona, United States; Arizona State University, Phoenix, Arizona, United States; Arizona State University, Phoenix, Arizona, United States; Arizona State University, Phoenix, Arizona, United States; Arizona State University, Phoenix, Arizona, United States

## Abstract

Hispanic/Latinos are 1.5x more likely to develop Alzheimer’s disease and related dementias (ADRD) than non-Hispanic/Latino Whites. As a result, there will be a corresponding increase in Hispanic/Latino family caregivers (H/LFC) needing resources to support them through the caregiving process. As of 2022, the Hispanic/Latino population only accounts for 2% of the clinical trials on ADRD or studies funded by the National Institute of Health. For H/LFC of people with ADRD, the limited outreach is due to eligibility requirements, time constraints, and language accessibility. This research examines the barriers to H/LFC outreach and identifies effective outreach strategies to engage them in interventions. Focus groups were conducted in English and Spanish in the Phoenix metropolitan area on H/LFC (n = 26, Mage = 51) and community health workers (n = 29, Mage = 46.5). Researchers employed a community engagement research methodology, holding three separate caregiver groups and three separate community health worker groups. Transcripts were analyzed inductively through thematic content analysis identifying three main categories: (1) Cultural Stigma on Dementia & Building Trust in Community Spaces, (2) Inadequate Spanish-Speaking Services & Enhancing Cultural Competencies, and (3) Systemic Inequities & Promotores-based Engagement Strategy. Each category identifies a barrier and a strategy. These findings provide insight into how researchers, clinicians, and community health workers can collaborate to find better strategies to recruit H/LFC into programs and clinical studies. It also offers opportunities to enhance retention efforts and maintain low attrition rates in not only clinical studies, but also family education and intervention programs.

